# Down-regulation of miR-9* in the peripheral leukocytes of Huntington’s disease patients

**DOI:** 10.1186/s13023-017-0742-x

**Published:** 2017-12-19

**Authors:** Kuo-Hsuan Chang, Yih-Ru Wu, Chiung-Mei Chen

**Affiliations:** Department of Neurology, Chang Gung Memorial Hospital Linkou Medical Center and College of Medicine, Chang Gung University, Taoyuan, Taiwan

**Keywords:** Huntington’s disease, Biomarker, MicroRNA, MiR-9*

## Abstract

**Background:**

Huntington’s disease (HD), caused by expansion of a polyglutamine tract within HUNTINGTIN (HTT) protein, is an autosomal dominant neurodegenerative disease associated with a progressive neurodegeneration of striatum and cerebral cortex. Although a few studies have identified substantial microRNA (miRNA) alterations in central nervous tissues from HD patients, it will be more accessible to employ these molecular changes in peripheral tissues as biomarkers for HD.

**Methods:**

We examined the expression levels of 13 miRNAs (miR-1, mirR-9, miR-9*, miR-10b, miR-29a, miR-29b, miR-124a, miR-132, miR-155, miR-196a, miR-196b, miR-330 and miR-615), 10 of which previously demonstrated alterations and 3 of which are potential regulators of differentially-expressed genes in brains of HD patients, in the peripheral leukocytes of 36 HD patients, 8 pre-symptomatic HD carriers and 28 healthy controls.

**Results:**

We found expression levels of miR-9* was significantly lower in HD patients compared with those in healthy controls, while other miRNAs did not show significant difference between these two groups. However, there was no significant correlation between Unified Huntington’s Disease Rating Scales (UHDRS) and levels of miR-9* in peripheral leukocytes of HD patients.

**Conclusion:**

Our findings indicate the potential of miR-9* in peripheral leukocyte as a signature of neurodegeneration in HD patients.

## Background

Huntington’s disease (HD) is an autosomal dominant neurodegenerative disease presented with psychiatric manifestations, cognitive decline, and choreiform movements [1]. A CAG trinucleotide repeat expansion in *HTT*, the causative gene mutation in HD, generates a polyglutamine tract in HUNTINGTIN (HTT) protein [1]. This polyglutamine tract leads to a conformational change in HTT and accumulation of insoluble intranuclear and intracytoplasmic aggregates. These abnormal aggregates may impair mitochondrial and proteosome pathways [2, 3], increase oxidative stress [3, 4], disrupt transcriptional regulation [5] and protein-protein interaction [6], and eventually lead to neuronal death particularly in the striatum and cortex [7]. Compelling evidence shows that these abnormal molecular pathway profiles may be detected not only in nervous but also in peripheral tissues [8–16]. And searching for biomarkers in peripheral tissues, especially from blood, is of high priority given the ease of sample collection.

MicroRNAs (miRNAs) are small non-coding RNAs that regulate gene expression at the post transcriptional level [17]. Binding of a miRNA to the 3′ untranslated regions (UTRs) of its target mRNA typically results in mRNA degradation and translational suppression [17]. Some miRNAs are highly expressed in the central nervous tissues [18] and appear to regulate vital neuronal function such as neuronal lineage commitment [19] and neurite outgrowth [20]. Recent studies have indicated altered expression patterns of miRNAs in neurodegenerative diseases including HD [21–24]. Hoss et al. identified five up-regulated miRNAs (miR-10b, miR-196a, miR-196b, miR-615 and miR-124a) in prefrontal cortex from HD patients [23]. Johnson et al. found reduced expression of miR-132 in the cortices of human HD patients, whereas miR 29a and miR-330 were up-regulated in the same human brain tissues [24]. Packer et al. found significant down-regulation of miR-9, miR- 9*, and miR-29b, as well as a significant dynamic change of miR-132 in the cortices of HD patients [22]. By applying network analysis for the gene expression datasets of HD post mortem brain regions, Neueder et al. predicted that upregulated gene expression modules involving DNA binding/zinc-finger and metallothionein in cerebellum is potentially regulated by miR-124, and upregulated gene expression modules involving amino acid catabolic process and protein folding/chaperones in frontal cortex are possibly regulated by miR-155 and miR-1, respectively [21]. Although the majority of miRNA profiling studies in HD have been performed in brain samples, miRNA dysregulations have been rarely found in other body fluids or tissues, such as plasma, peripheral blood and cerebrospinal fluid. For example, HD gene carriers demonstrate high level of miR-34a in plasma before the onset of clinical manifestations [25]. Herein we chose 10 miRNAs that previously demonstrated alterations in the brains of HD patients [22–24] and 3 miRNAs that are potential regulators of differentially-expressed genes in brains of HD patients to examine their expression in the peripheral leukocytes of HD patients and gender- and age-matched healthy controls (HCs).

## Methods

### Ethics statement and study populations

The diagnosis of HD patients and pre-symptomatic HD (preHD) carriers was confirmed by a neurological and genetic test showing expanded CAG repeats in the exon 1 region of the *HTT* [1]. Unified Huntington’s Disease Rating Scale (UHDRS) were recorded for each patient [26]. Patients with liver or renal dysfunctions, cardiac and pulmonary disease, infection, malignancy or pregnancy were excluded. This study was approved by the Institutional Review Boards of Chang Gung Memorial Hospital and all the individuals signed a written informed consent.

#### Sample collection

Blood samples were collected into PaxgeneTM blood RNA tube (Pre-AnalytiX, Qiagen, Valencia, CA). Total RNA of leukocytes was extracted using the PaxgeneTM blood RNA Extraction Kit (Pre-AnalytiX, Qiagen, Valencia, CA), and transferred into the RNeasy MinElute spin column (RNeasy MinElute Cleanup Kit, Qiagen, Valencia, CA) according to manufacturer’s instruction. RNA quality was determined by the A260/A280 absorption ratio.

#### Quantification of miRNA expression in peripheral leukocytes

RNA was converted to cDNA using the SuperScript III First-Strand (Invitrogen). For each sample, miRNA expression levels were quantified using the TaqMan miRNA assays (miR-1, mirR-9, miR-9*, miR-10b, miR-29a, miR-29b, miR-124a, miR-132, miR-155, miR-196a, miR-196b, miR-330, miR-615 and RNU48) and the ABI 7900HT Sequence Detection System Applied Biosystems). Each reaction included cDNA from 100 ng of RNA, 900 nM of each primer, 100 nM of probes or primers and Universal PCR Master Mix (Applied Biosystems). PCR parameters were 50 °C for 2 min, 95 °C for 10 min, 40 cycles of 95 °C for 15 s, 60 °C for 1 min. Each sample was assessed in duplicate. Cycle threshold (CT, the fractional cycle number where the fluorescent signal reaches detection threshold) in each reaction was set in the linear range. Relative expression values were normalized to RNU48. Relative gene expressions were calculated using the 2^ΔCT^ method, ΔCT = CT (RNU48) – CT (target miRNA). For each set of values, data were expressed as means ± standard deviation (SD). Differences between groups were evaluated by analysis of variance (ANOVA) with *post*-*hoc* Bonferroni test or analysis of covariance (ANCOVA) adjusted by age and gender where appropriate. Correlations of UHDRS (motor scale, independence scale and functional capacity), size of expanded CAG repeats or disease duration with levels of mRNA were analyzed by covariate-adjusted generalized linear model (adjusted by age and gender). All *P*-values were two-tailed. The values of *P* < 0.05 were considered significant.

## Resultss

Previous report showed that the expression levels of a number of miRNAs, including mirR-9 [22], miR-9* [22], miR-10b [23], miR-29a [24], miR-29b [22], miR-132 [24], miR-196a [23], miR-196b [23], miR-330 [24], miR-615 [23], were altered in brain samples of HD patients. The mir-1, miR-124a, and miR-155 were the potential regulators that were predicted to control the differentially-expressed genes in brains of HD patients [21]. Here we measure the expression levels of above miRNAs in peripheral leukocytes of a cohort including 36 HD patients, 8 PreHD carrier and 28 HCs (Table 1). The age of preHD carriers (29.75 ± 7.21 years) was significantly younger than HCs (42.03 ± 11.01 years) and HD patients (45.58 ± 12.53 years), whereas the age of HD patients is not different from that of the HCs.

**Table 1:** Demographic characteristics and blood biochemical parameters of healthy controls and the patients with Huntington’s disease (HD)

	HD (*n* = 36)	PreHD (*n* = 8)	Healthy controls (*n* = 28)
Age (years)	45.58 ± 12.53	29.75 ± 7.21^*^	42.03 ± 11.01
Male (%)	20 (55.56)	3 (37.50)	17 (58.62)
HD duration (years)	4.38 ± 3.09		
Expanded CAG repeat number	46.42 ± 9.07	44.13 ± 3.17	
Drugs (%)			
Dopamine antagonist	18 (50.00)	0 (0)	0 (0)
Anti-depressants (SSRI, SNRI, NaSSA)	9 (25.00)	0 (0)	0 (0)
Amantadine	5 (13.89)	0 (0)	0 (0)
UHDRS			
Motor score	27.2 ± 18.94	0	
Independence scale	78.57 ± 21.98	100	
Functional capacity	9.31 ± 3.71	13	

Scale ranges (normal to most severe) include motor score (0 to 124), independence scale (100 to 10), and functional capacity (13 to 0)

*HD* Huntington’s Disease, *PreHD* pre-sympatomatic Huntington’s disease, *NaSSA* Noradrenergic and specific serotonergic antidepressant, *SNRI* Serotonin–norepinephrine reuptake inhibitor, *SSRI* Selective serotonin re-uptake inhibitors, *UHDRS* The Unified Huntington’s Disease Rating Scale

^*^Statistically significant in comparison with HD patients and healthy controls respectively, *P* < 0.05, ANOVA with *post-hoc* Bonferroni test

HD patients displayed significantly lower expression level of miR-9* (0.0008 ± 0.0013) compared with HCs (0.0028 ± 0.0035, *P* = 0.005, Table 2). With the estimated standard deviations in each marker, at the level of 0.05, present sample sizes achieve a power of 81.95% for miR-9* to detect differences in the mean between HD and HC. MiR-9* plays an important role in regulating expression of neuron-specific genes [22, 27, 28]. Its down-regulation may be relevant to the pathogenesis of HD and may serve a potential therapeutic target for HD. On the other hand, we calculated the correlation between miR-9* expression level and age, medications, or UHDRS by covariate-adjusted generalized linear model. All factors did not show significant correlation with expression levels of miR-9* (data not shown). Given that our sample size is relatively small, large cohort studies will be warranted to clarify if miR-9* may serve as a biomarker to monitor the disease status in HD.

**Table 2:** miRNA levels in the peripheral leukocytes from patients with Huntington’s disease versus healthy controls

miRNA	HD (*n* = 36)	PreHD (*n* = 8)	Normal control (*n* = 28)	*P* value^‡^
miR-9^*^	0.0008 ± 0.0013^*^	0.0008 ± 0.0011	0.0028 ± 0.0035	0.005
miR-9	0.0019 ± 0.0019	0.0007 ± 0.0008	0.0037 ± 0.0055	0.064
miR-1	0.0013 ± 0.0015	0.0018 ± 0.0022	0.0029 ± 0.0035	0.070
miR-330	0.0128 ± 0.0081	0.0208 ± 0.0157	0.0184 ± 0.0098	0.071
miR-29b	0.0023 ± 0.0043	0.0055 ± 0.0061	0.0038 ± 0.0039	0.075
miR-10b	0.0013 ± 0.0012	0.0024 ± 0.0029	0.0022 ± 0.0019	0.092
miR-615	0.0238 ± 0.0370	0.0080 ± 0.0074	0.0336 ± 0.0328	0.212
miR-196a	0.0072 ± 0.0063	0.0136 ± 0.0143	0.0122 ± 0.0139	0.252
miR-196b	0.0089 ± 0.0093	0.0146 ± 0.0156	0.0161 ± 0.0213	0.265
miR-155	0.0684 ± 0.0505	0.0928 ± 0.0765	0.0639 ± 0.0464	0.448
miR-132	0.0381 ± 0.0255	0.0476 ± 0.0346	0.0457 ± 0.0354	0.661
miR-29a	0.0926 ± 0.1003	0.1024 ± 0.0911	0.1136 ± 0.1368	0.841
miR-124a	Not detectable	Not detectable	Not detectable	

*HD* Huntington’s Disease, *PreHD* pre-sympatomatic Huntington’s disease

^‡^ *P* value of ANCOVA with adjustment of age and gender

^*^Statistically significant in comparison with healthy controls, *P* < 0.05, ANCOVA with adjustment of age and gender

Regarding to other miRNAs, the expression level of miR-124a was not detectable in peripheral leukocytes. The expression levels of the rest of miRNAs were similar in HD patients, PreHD carrier and HCs.

## Discussion

Given that a central nervous tissue sample from HD patients is difficult to obtain, a biomarker in peripheral tissues, especially from blood, should be more feasible to indicate disease status. Although the main pathology of HD is in the striatum, some studies have detected parallel biochemical changes in peripheral tissues [29–33]. Immune activation [29], oxidative damage to DNA [30], altered activation of Akt pathway [31], reduction of cAMP [32] and creatinine kinase-BB [33] have been demonstrated in both peripheral blood and brain of HD patients or mouse models. Here we found the expression level of miR-9* in peripheral leukocytes was significantly lower in HD patients compared with that HCs. The down-regulation of miR-9* may be involved in the pathogenesis of HD.

A single miR-9 precursor produces two mature miRNAs, miR-9 and miR-9*, which are abundantly expressed in both developing and adult brain [34]. MiR-9 is highly conserved across vertebrate species and shows brain-specific expression [35, 36]. The complementary strand miR-9* is also crucial for brain development [27, 28, 37]. MiR-9* may control the transition from neural progenitor to postmitotic neurons by switching the chromatin remodeling complexes from neural-progenitor-specific Brg/Brm-associated factor (npBAF) to neuron-specific BAF [27, 28]. Suppressing miR-9* activity in postmitotic neurons induces the expression of BAF53a, which is an npBAF [27]. Overexpression of miR-9* with miR-9 and miR-124 converts human fibroblasts into neurons [28], indicating its role in neuronal differentiation. In adult brains, the expression levels of miR-9* are decreased in neurodegenerative diseases such as HD and Alzheimer’s disease [22, 38]. Inhibition of miR-9* activity affects hippocampus-dependent memory by suppressing hippocampal long-term potentiation (LTP) and regulating LTP-related genes *DMD* and *SAP97* [39]. These findings suggest that miR-9*-mediated gene regulation is important for synaptic plasticity and memory, both of which play an important role in the generation of abnormal movements and psychiatric abnormalities in HD.

MiR-9* also demonstrates the potential of regulating the expression of co-repressor of repressor element 1-silencing transcription factor (CoREST) that contains predicted MiR-9* regulatory site [22]. In neurons, repressor element 1-silencing transcription factor (REST) is expressed at low level and mainly confined to the cytoplasm by binding to normal HTT [40]. However, the binding of mutant HTT to REST reduces, which allows REST to translocate to nucleus [40]. In nucleus, REST down-regulates expression of neuron-specific genes by recruiting co-repressors CoREST and binding to repressor element 1 (RE1) consensus sequences [40, 41]. These genes suppressed by REST and CoREST encode a number of proteins involved in maintaining function of cholinergic, GABAergic and glutaminergic neurons and in differentiation of medium spiny projection neurons [42]. For example, BDNF expression is down-regulated in brain of HD due to increased binding of REST to RE1 site of BDNF promoter [40]. The down-regulation of miR-9* may therefore increase expression of CoREST, leading to down-regulation of specific neurotrophic genes targeted by CoREST, which may mediate the selective neuronal dysfunction in striatum of HD. On the other hand, RE1 sequence is also located in close proximity to miR-9* and REST may bind to the RE1 site to suppress the expression of miR-9* [43]. Since mutant HTT tends to dissociate from REST, the released REST is more likely to occupy RE1 site in the promoter to suppress miR-9* expression. The decreased miR-9* expression results in increased CoREST, which would further down regulate miR-9* in a vicious cycle in HD.

The limitation of the study is that the small sample size, especially for the preHD group, may lower the statistical power. Another general issue is the differences in the baseline characteristics of the study groups, such as age and gender distribution, although are small, which may affect the results of our study. Some unknown interactions of nutrition and medications may partially contribute to the regulation of miRNA expression between groups.

## Conclusion

Our results clearly demonstrate the alterations of miRNA levels in peripheral leukocytes of HD patients, which has not been reported before. A larger, multi-center, longitudinal study regarding the correlation of peripheral miRNA expression levels with clinical and neuroimaging features will determine the potential application of peripheral miR-9* as biomarkers for HD, and might shed light on the involvement of miR-9* in HD.

## Abbreviations

CoREST: Co-repressor of repressor element 1-silencing transcription factor
HC: Healthy control
HD: Huntington’s disease
HTT: Huntingtin
LTP: Long-term potentiation
MiRNA: microRNA
npBAF: Neural-progenitor-specific Brg/Brm-associated factor
PreHD: Pre-symptomatic Huntington’s disease
RE1: Repressor element 1
REST: Repressor element 1-silencing transcription factor
UHDRS: Unified Huntington’s Disease Rating Scale
UTR: Untranslated regions
